# Novel polymerase spiral reaction assay for the visible molecular detection of porcine circovirus type 3

**DOI:** 10.1186/s12917-019-2072-9

**Published:** 2019-09-06

**Authors:** Jun Ji, Xin Xu, Xueyu Wang, Kejing Zuo, Zhili Li, Chaoliang Leng, Yunchao Kan, Lunguang Yao, Yingzuo Bi

**Affiliations:** 1 Henan Provincial Engineering Laboratory of Insect Bio-reactor and Henan Key Laboratory of Ecological Security for Water Source Region of Mid-line of South-to-North, Nanyang Normal University, 1638 Wolong Road, Nanyang, Hena, 473061 People’s Republic of China; 2 Veterinary Laboratory, Guangzhou Zoo, Guangzhou, 510642 People’s Republic of China; 3 College of Animal Science, South China Agricultural University, Guangzhou, 510642 People’s Republic of China

**Keywords:** Molecular detection, PCV3, Polymerase spiral reaction, Visible detection

## Abstract

**Background:**

Porcine circovirus type 3 (PCV3) is a newly emerging circovirus that might be associated with porcine dermatitis and nephropathy syndrome, reproductive failure, and cardiac and multisystemic inflammation. To aid the prevention and control of the infectious disease caused by PCV3, we developed a novel isothermal amplification assay using polymerase spiral reaction (PSR), which allows the visual detection of preserved strains and clinical samples.

**Results:**

This assay precisely amplified the PCV3 genome with the use of a water bath at 62 °C for 50 min. The detection limit was found to be 1.13 × 10^2^ copies/μL by gel electrophoresis or with the use of a visible dye (an indicator comprising phenol red and cresol red). No cross-reaction with other porcine infectious viruses was observed. The detection results for 23 PCV3-positive samples by PSR were in accordance with loop-mediated isothermal amplification (LAMP) assay. The detection rate of the PSR assay for PCV3 positivity of clinical samples was 68/97, which was higher than LAMP assay (67/97).

**Conclusions:**

These results indicated that the PSR assay provides an accurate and rapid method for the detection of PCV3 with high sensitivity and specificity. It is particularly suited for use in a simple laboratory setting without a thermal cycler or gel electrophoresis equipment.

## Background

Porcine circovirus type 3 (PCV3) is a newly emerging virus associated with porcine dermatitis and nephropathy syndrome, reproductive failure, and multiorgan inflammation, even though its causative role in any of these syndromes has yet to be demonstrated [1–3]. The virus was first reported in pig farms in the USA in 2016 through metagenomic sequencing and was identified to be a new member of the genus *Circovirus* of the family *Circoviridae* [4]*.* This genus also includes porcine circovirus type 1, which has no clinical manifestations, and porcine circovirus type 2 (PCV2), which has proved to be a significant economic threat for the pig industry [5, 6]. Other reports indicated that PCV3 has already been circulating in pig-producing countries for some time before its first detection, although not in the USA, with increasing numbers of infectious cases being reported in Italy, Brazil, Germany, South Korea, and China [7–12]. In addition, there is accumulating evidence that coinfections of PCV3 with other pathogens may be associated with increased pathogenicity in pigs [1, 13].

Conventional serological methods have been used to identify PCV3. These methods include an enzyme-linked immunosorbent assay that can accurately detect the viruses, but it can be time-consuming [14, 15]. Molecular methods based on polymerase chain reaction (PCR) assays, both routine and quantitative, have been developed to monitor and detect PCV3 rapidly and specifically [16–18]. However, these methods require sophisticated thermal cyclers, thereby limiting their usefulness for the practical point-of-care testing of clinical samples. To avoid the need for professional laboratory equipment, isothermal molecular methods for the detection of PCV3 have been developed, including methods based on loop-mediated isothermal amplification (LAMP) and recombinase polymerase amplification (RPA) [19, 20]. These isothermal methods have the potential to differentiate PCV3 from other pathogens with high specificity, but neither technique is without imperfections. The LAMP assay for PCV3 detection requires four primers to target at least six sequence regions, strict primer coordination, and a more conserved sequence [20, 21]. The recombinase polymerase amplification technique needs only one primer pair to accomplish the detection course; however, its reaction products cannot be determined directly by the naked eyes [19].

Polymerase spiral reaction (PSR) is a novel assay technique that requires only one pair of primers, making this assay easier to design and more cost-effective than LAMP assays [22]. Furthermore, the reaction results include a high level of pyrophosphate ion byproducts, which can be directly visualized by adding a suitable pH indicator. Building on these advantages, we have developed a novel PSR assay for the detection of PCV3. Here we describe the assay and evaluate its accuracy through the detection of clinical samples.

## Results

### Optimal reaction temperature and time for the PCV3 PSR assay

In the agarose gel electrophoresis analyses, a little difference displayed between the reactions from 60 °C to 65 °C; however, the PSR products at 62 °C displayed the brightest bands. At this temperature, the band reached its maximum brightness at 50 min. Thus, the optimal PCV3 PSR amplification reaction conditions were found to be using a water bath at 62 °C for 50 min.

### Sensitivity test results

**Fig. 1:**
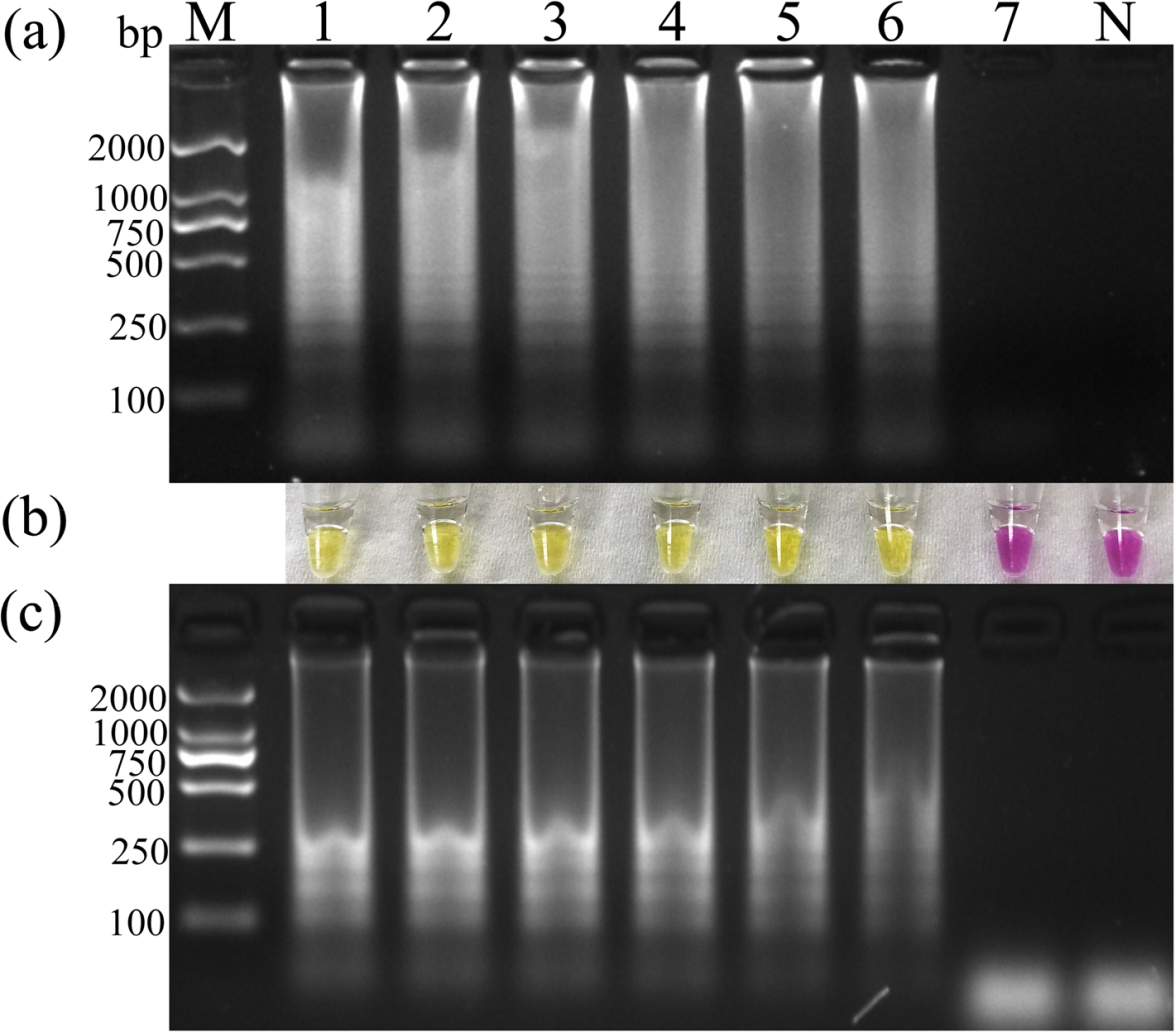
Sensitivity of the polymerase spiral reaction (PSR) and loop mediated isothermal amplification for the detection of Porcine circovirus type 3 (PCV3). Ten-fold serial dilutions of standard plasmid (PCV3-ORF2) were subjected to the PSR and LAMP assays and analyzed. Lane M, molecular size marker DL2000. Lanes 1–7, dilutions of PCV3-*ORF*2 (1.13 × 10^7^ copies, 1.13 × 10^6^ copies, 1.13 × 10^5^ copies, 1.13 × 10^4^ copies, 1.13 × 10^3^ copies, 1.13 × 10^2^ copies, and 1.13 × 10^1^ copies); and lane N, negative control. **a** Agarose gel electrophoresis demonstrating the sensitivity of the PSR assay. **b** Colorimetric analysis demonstrating the sensitivity of the PSR assay. **c** Agarose gel electrophoresis demonstrating the sensitivity of the LAMP assay

The sensitivities of the PSR and LAMP assays were compared by detecting their reaction products in agarose gel electrophoresis (Fig. 1). The positivity of the PSR products was also determined by adding a visible dye and observing the color change by the naked eye. The three runs were 100% coincident. Therefore, the detection limits of the PSR and LAMP assays were both 1.13 × 10^2^ copies/μL. In the visible sensitivity analysis, the color of the dye changed from purple–red to yellow at the same detection limit as that determined by agarose gel electrophoresis.

### Specificity of the PCV3 PSR assay

**Fig. 2:**
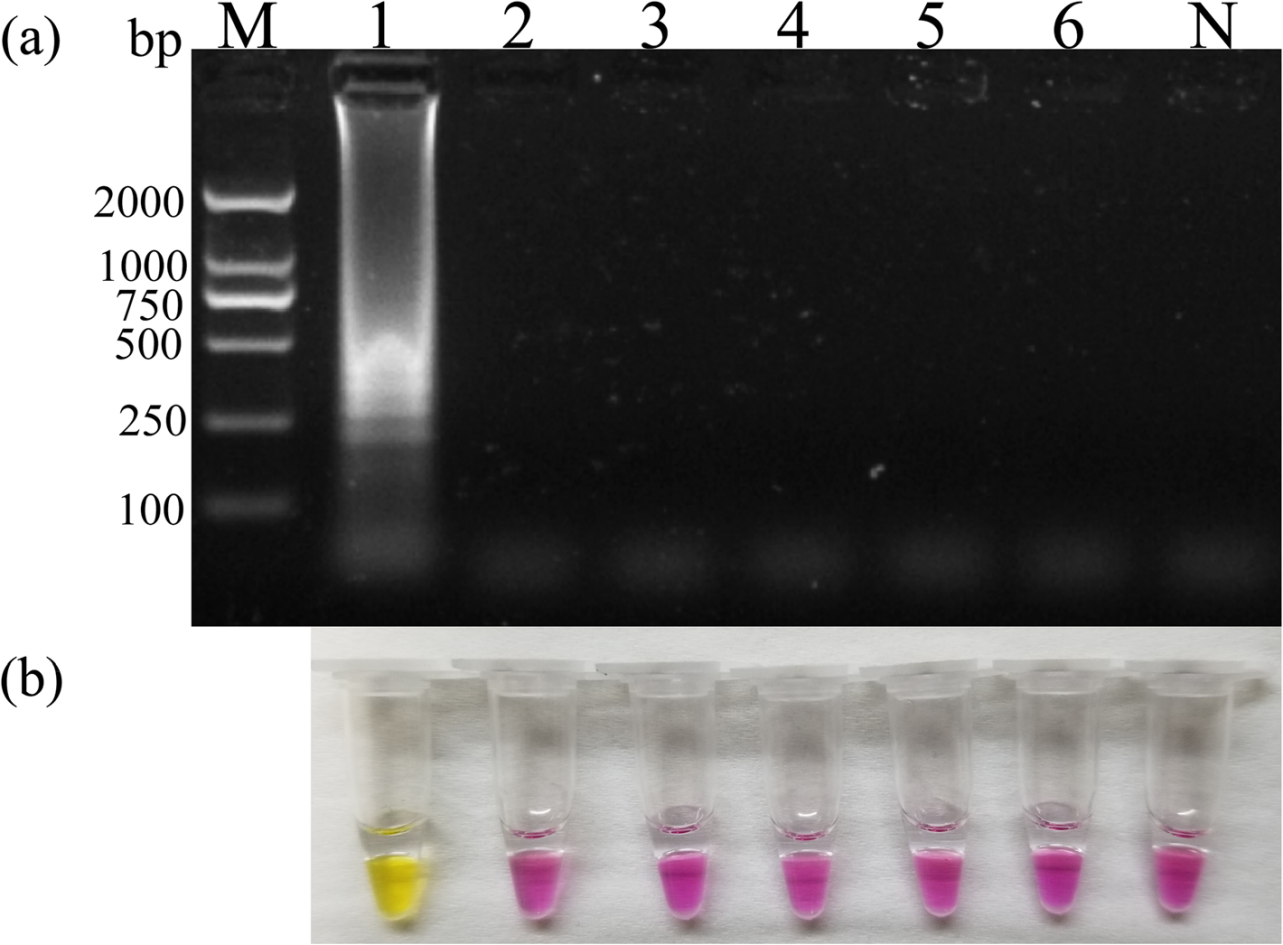
Determination of PSR specificity. Lane M, molecular size marker DL2000; lane 1, PCV3; lane 2, pseudorabies virus (PRV); lane 3, porcine reproductive and respiratory syndrome virus (PRRSV); lane 4, porcine circovirus type 2 (PCV2); lane 5, classical swine fever virus (CSFV); lane 6, porcine epidemic diarrhea virus (PEDV); and lane N, negative control. **a** Specificity of the PSR assay as determined by electrophoretic separation of the reaction products. **b** Colorimetric analysis of the reactions demonstrating the specificity of the PSR assay

The specificity test confirmed that the PSR assay was specific for PCV3; only PCV3 positive samples showed clear strip-bands, whereas samples positives to other viruses (pseudorabies virus (PRV), porcine reproductive and respiratory syndrome virus (PRRSV), porcine circovirus type 2 (PCV2), classical swine fever virus (CSFV), porcine epidemic diarrhea virus (PEDV) did not. The results were in good agreement with colorimetric analysis of the reactions (Fig. 2), confirming the good specificity of the PCV3 PSR assay.

### Application of the PCV3 PSR assay to clinical samples

There was absolute agreement between the LAMP and PSR assays for the detection of the 23 positive samples. For the clinical tissue samples, the detection rate for PCV3 positivity was as follows: PSR, 68/97 and LAMP, 67/97. The detailed detection results for the clinical samples are listed in Table 1.

**Table 1:** Comparison of the detection of PCV3 in clinical samples by polymerase spiral reaction (PSR) and loop-mediated isothermal amplification (LAMP)

Province	Date	Tissues	Detection positive rate for different assay	
			LAMP	PSR
Jiangsu	2017.11	spleen	15/17^a^	15/17
Hunan	2018.01	pooled liver and spleen	13/19	13/19
Hubei	2018.02	spleen	21/24	21/24
Hubei	2018.03	spleen	6/11	6/11
Hunan	2018.04	pooled liver and spleen	4/14	5/14
Anhui	2018.05	spleen	8/12	8/12

^a^The two figures indicate the number of positive results/the number of samples

## Discussion

Increasing number of reports have suggested that the PSR technique provides a promising isothermal DNA amplification assay that can be used for rapid and low-resource diagnostics, including for detecting some human pathogens and African swine fever virus [23–26]. Cooperated with reverse transcriptase, one-step reverse transcription polymerase spiral reaction (RT-PSR) assay could be used for the rapid detection of porcine epidemic diarrhea virus (PEDV) [27]. In this study, the PCV3 PSR assay displayed similar detection limit with the LAMP assay; it also displayed remarkable accuracy with good specificity for positive samples. The PCV3 PSR assay was most effective at 62 °C; however, the products amplified at all the temperatures tested (61 °C–65 °C) displayed remarkable results on agarose gel electrophoresis, making this assay practical for use in basic laboratories with low-precision incubating equipment, such as water baths. The reaction time of the PCV3 PSR assay was approximately only half of the time required for conventional PCR and other PCR-based methods. The visible analysis method for the PSR reaction using a pH indicator without electrophoresis and gel imaging could additionally save time.

A recombinase polymerase amplification (RPA) method combined with a lateral flow strip has recently been developed for visible detection; however, use of the lateral flow strip increases costs and procedure time [28]. Using SYBR Green I dye with the LAMP assay for PCV3 detection can satisfactorily reveal dissimilarities between positive and negative tubes; however, the dye must be added at the end of the reaction for the inhibitory effect. In these methods, the operations needed for reaction with the lateral flow strip or to add the SYBR Green I dye could increase the risk of false positive results due to aerosol contamination by amplified products [20, 28]. Similarly, the high sensitivity of PSR may also contribute to its susceptibility to false-positive results because of the carryover or cross-contamination in the reaction system. Therefore, we using the phenol red dye added before the reaction could reduce aerosol contamination to some extent. To minimize contamination and false-positive results from PSR in practice, sample preparation and PSR reaction and electrophoretic run should be carried out in isolated places. In the detection comparison performed on 97 clinical samples, the PSR assay detected 68 samples positive for PCV3 DNA, whereas the LAMP assays detected67 positive samples, respectively. The PSR assay did not detect PCV3 in 29 of the samples; this was in agreement with the results of LAMP assays. One sample from the Hunan Province was detected as negative by the LAMP assay but was detected as positive by the PSR assays. This missed detection may have been because of a mismatch of primers, suggesting that the PCV3 is undergoing constant evolution and variant. This shows that PCV3 monitoring requires continuous investigation and primer updates. It also demonstrates the practicality of the PSR assay for the clinical detection of PCV3; the assay requires only one pair of primers, making it easier to design when there are mismatches. The high detection rate again confirmed the accuracy and sensitivity of the PCV3 PSR assay.

## Conclusions

In conclusion, this report describes a novel diagnostic test for PCV3 that is deployable in the field. This PSR assay could be developed into a rapid and reliable diagnostic method with the potential for routine use for the detection of PCV3.

## Methods

### Samples

From winter 2017 to spring 2018, spleens and liver tissues of dead pigs suspected to have been infected with PCV3 were collected from farms of Jiangsu, Hunan, Hubei and Anhui province of China. The samples were washed with phosphate-buffered saline (PBS) and immersed in liquid nitrogen prior to grinding. A 20% suspension was prepared by adding PBS and then repeatedly freezing and thawing three times. The pretreated tissues were centrifuged at 5000 rpm for 10 min, and 0.2 mL of the supernatant was taken and maintained at − 80 °C prior to nucleic acid extraction.

### Extraction of viral DNA and RNA

Viral DNA and RNA were extracted from the samples with a DNA/RNA extraction kit (TransGen Biotech, Beijing, China), according to the manufacturer’s instructions. The purity and concentrations of the DNA and RNA were determined by a biological spectrophotometer. The samples were stored at − 20 °C until use.

### Primers design

Primers used for the PSR assay were designed according to the conserved regions of the *ORF2* gene of PCV3 using Oligo v7.37 (Molecular Biology Insights Inc., Colorado Springs, CO, US). The specificity of primers was preliminarily determined by using NCBI-Blast (https://blast.ncbi.nlm.nih.gov/Blast.cgi). The primers used for amplification efficiency after the initial screening are shown in Table 2.

**Table 2:** Primer sets for the polymerase spiral reaction assays (The lower-case 5′ sequence of the forward primer (SF) abstracted from a botanic gene is reverse to the lowercase 5′ sequence of the reverse primer (SR) [22])

Primer name	Sequence 5′-3′	Position within PCV3-*ORF*2 ^a^
SF	acgaattcgtacatagaagtatagGTCTTGGAGCCAAGTGTTTGTG	277–299
SR	gatatgaagatacatgcttaagcaCTTCATTACCCGCCTAAACGAG	433–453

^a^The primer position is based on the sequence of the Chinese Hubei-610/2016 strain, GenBank accession number: KY354038

### Establishing the reaction conditions for PSR

Based on the optimal constituents identified in previous tests, the PSR reaction was performed in a volume of 25 μL containing each primer (forward primer PCV3-SF and backward primer PCV3-SR, both 0.8 μM), a 1.2 mM mixture of dNTPs, 0.8 M betaine, 6 mM MgSO_4_, 8 U of *Bst* DNA polymerase 3.0 (New England Biolabs, Ipswich, MA, USA), 10 mM (NH4)_2_SO_4_, 50 mM KCl, 0.1% v/v Tween-20, and 1 μL of template DNA. For the colorimetric analysis of the products, 1 μL of pH-sensitive dye (comprising 0.025 mM phenol red and 0.08 mM cresol red) was added to each tube. In addition, 25 μL of mineral oil was added to prevent the volatilization of the PSR products. The PSR products with repeat target sequences were produced due to different spiral amplification stages as a result of simultaneous Bst DNA polymerase extension at 3′ end and strand displacement at 5′ end [22]. To establish the optimal reaction temperature and time the for PSR assay, gradient optimization was performed first at reaction temperatures of 60 °C, 61 °C, 62 °C, 63 °C, 64 °C, and 65 °C for 60 min, and then for 30, 40, 50, and 60 min at the optimized temperature. For each temperature and time, the reaction product was observed by electrophoresis on 2% agarose gel, the optimal reaction time and temperature were determined according to the clarity and brightness of the obtained bands.

### Sensitivity of the PCV3 PSR assay

The 645-bp *ORF2* gene from a Chinese PCV3 strain was amplified and cloned into the commercial clone vector pMD 18-T (TaKaRa Biotech Corporation, Dalian, China) to create a standard plasmid (PCV3-ORF2). This was used to compare the sensitivities of the PSR and LAMP assays. The limits of detection of the two methods were evaluated by serial 10-fold dilutions of the standard template of the PCV3-ORF2 plasmid from 1.13 × 10 to 1.13 × 10^7^ copies/μL, and performed three times to properly assess the detection limit of the two isotherm methods. The LAMP reaction system (25 μL) included the same primer sets as reported and 8 U of *Bacillus stearothermophilus* DNA polymerase. It was performed by using a water bath at 60 °C for 60 min, as described previously [20]. The amplified DNA product was identified using 2% agarose gel.

### Specificity of the two assays

The specificity of the PCV3 PSR primers was evaluated by applying the assay to positive samples for reference porcine viruses of a range of DNA types, and ddH_2_O was used as a negative control. These included DNA extracted from PCV2 and PRV and complementary DNA converted from the RNA of CSFV, PRRSV and PEDV using a PrimeScript™ RT Reagent Kit with a gDNA Eraser Kit (TaKaRa Biotech Corporation, Dalian, China).

### Validation with clinical samples

The diagnostic accuracy of the PCV3 PSR assay was evaluated using viral DNAs of 23 PCV3-positive samples and 97 clinical samples collected from various farms located in Jiangsu, Anhui, Hubei, and Hunan provinces in central China. These samples were analyzed by PSR and LAMP assays simultaneously. The positive detection rates of the two methods were calculated and compared using LAMP as a gold standard.

## Data Availability

The datasets used and/or analyzed during the current study are available from the corresponding author on reasonable request.

## Abbreviations

CSFV: classical swine fever virus
LAMP: loop-mediated isothermal amplification;
PCR: polymerase chain reaction
PCV2: porcine circovirus type 2
PCV2: porcine circovirus type 3
PEDV: porcine epidemic diarrhea virus
PRRSV: porcine reproductive and respiratory syndrome virus
PRV: pseudorabies virus
PSR: polymerase spiral reaction
RPA: recombinase polymerase amplification
